# Propensity Score Matched Comparison of Intensity Modulated Radiation Therapy vs Stereotactic Body Radiation Therapy for Localized Prostate Cancer: A Survival Analysis from the National Cancer Database

**DOI:** 10.3389/fonc.2017.00185

**Published:** 2017-08-31

**Authors:** Anthony Ricco, Alexandra Hanlon, Rachelle Lanciano

**Affiliations:** ^1^Philadelphia Cyberknife, Havertown, PA, United States; ^2^Crozer Keystone Health Care System, Springfield, PA, United States; ^3^University of Pennsylvania, School of Nursing, Philadelphia, PA, United States

**Keywords:** stereotactic body radiation therapy, intensity modulated radiation therapy, prostate cancer, National Cancer Database, overall survival

## Abstract

**Purpose:**

No direct comparisons between extreme hypofractionation and conventional fractionation have been reported in randomized trials for the treatment of localized prostate cancer. The goal of this study is to use a propensity score matched (PSM) analysis with the National Cancer Database (NCDB) for the comparison of stereotactic body radiation therapy (SBRT) and intensity modulated radiation therapy (IMRT) for organ confined prostate cancer.

**Methods:**

Men with localized prostate cancer treated with radiation dose ≥72 Gy for IMRT and ≥35 Gy for SBRT to the prostate only were abstracted from the NCDB. Men treated with previous surgery, brachytherapy, or proton therapy were excluded. Matching was performed to eliminate confounding variables *via* PSM. Simple 1–1 nearest neighbor matching resulted in a matched sample of 5,430 (2,715 in each group). Subset analyses of men with prostate-specific antigen (PSA) > 10, GS = 7, and GS > 7 yielded matched samples of 1,020, 2,194, and 247, respectively.

**Results:**

No difference in survival was noted between IMRT and SBRT at 8 years (*p* = 0.65). Subset analyses of higher risk men with PSA > 10 or GS = 7 histology or GS > 7 histology revealed no difference in survival between IMRT and SBRT (*p* = 0.58, *p* = 0.68, and *p* = 0.62, respectively). Variables significant for survival for the matched group included: age (*p* < 0.0001), primary payor (*p* = 0.0001), Charlson/Deyo Score (*p* = 0.0002), PSA (*p* = 0.0013), Gleason score (*p* < 0.0001), and use of hormone therapy (*p* = 0.02).

**Conclusion:**

Utilizing the NCDB, there is no difference in survival at 8 years comparing IMRT to SBRT in the treatment of localized prostate cancer. Subset analysis confirmed no difference in survival even for intermediate- and high-risk patients based on Gleason Score and PSA.

## Summary

The use of extreme hypofractionation using stereotactic body radiation therapy (SBRT) for localized prostate cancer remains controversial. There are no current randomized controlled trials comparing SBRT for localized prostate cancer with the current standard, intensity modulated radiation therapy (IMRT). Using the National Cancer Database with propensity score matching, we demonstrate no survival difference between SBRT and IMRT, including subset analysis of intermediate- and high-risk patients.

## Introduction

Intensity modulated radiation therapy (IMRT) is a standard radiation modality used in the treatment of organ confined prostate cancer. Ten-year actuarial data (median follow-up of 8 years) is available for high-dose IMRT up to 81 Gy which demonstrates high efficacy in preventing biochemical failure with acceptable side effects (1). Stereotactic body radiation therapy (SBRT) has been accepted as an “appropriate alternative for select patients with low to intermediate-risk disease” as per the ASTRO policy update of April 2013 and is also supported by the National Comprehensive Cancer Network (NCCN). SBRT publications have validated freedom from biochemical failure (FFBF) with up to 9-year actuarial data (median follow-up of 7 years) and side effect rates comparable with IMRT (2–4).

The combination of prostate cancer’s low a/b ratio, known benefit of dose-escalation, and efficacy/safety of high-dose rate brachytherapy led to single institutional, multi-institutional, and randomized clinical trials of SBRT for the treatment of prostate cancer (5, 6). Randomized data are lacking comparing the outcome of treatment for SBRT compared with IMRT for localized prostate cancer. The primary goal of this study is to compare survival between SBRT and IMRT for men with organ confined prostate cancer utilizing the National Cancer Database (NCDB).

## Materials and Methods

The NCDB is jointly sponsored by the American College of Surgeons and the American Cancer Society. It is a clinical oncology database sourced from hospital registry data collected in more than 1,500 commission on cancer-accredited facilities. NCDB data are used to analyze and track patients with malignant neoplastic diseases, their treatments, and outcomes. Data represent approximately 70% of newly diagnosed cancer cases nationwide and 30 million historical records. The NCDB includes prostate cancer patients treated from 2004 to 2013 providing information on demographics, risk factors specific to prostate cancer, staging information, treatment, and survival data. Patients are de-identified and the database is then sent to individual researchers for analysis after application and acceptance for individual projects.

**Figure 1:**
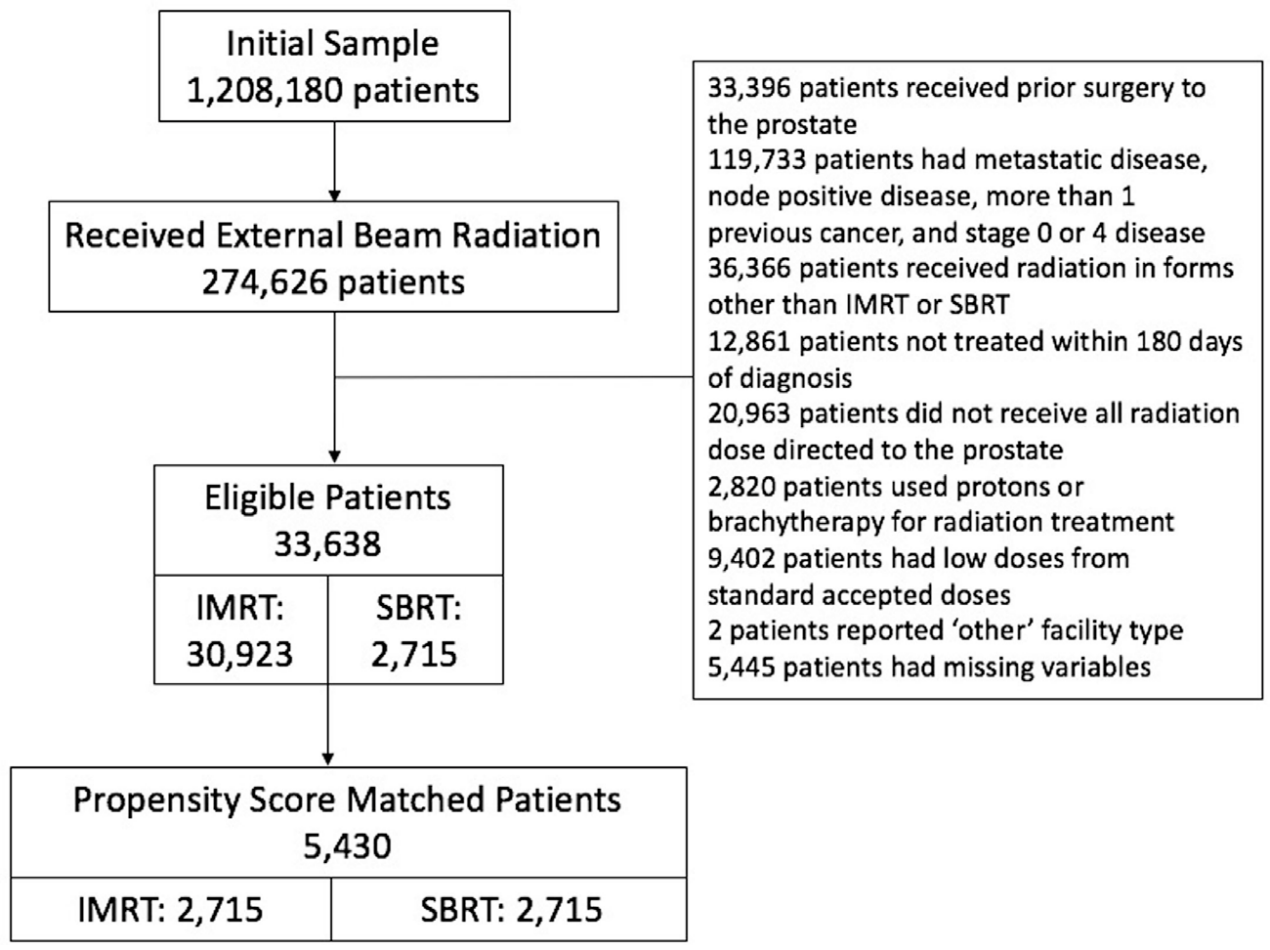
CONSORT diagram.

We initially identified 274,626 patients who received external beam radiation of some form. We excluded those patients who received prior surgery to the prostate. We excluded all but those patients who were listed as invasive adenocarcinoma of the prostate. We excluded those patients with metastatic disease, node positive disease, more than one previous cancer, and stages 0 and 4 disease. Of those, we excluded patients who received radiation in forms other than IMRT or SBRT. We included all patients diagnosed between 2004 and 2013 and treated within 180 days of diagnosis to rule out patients on active surveillance. We included only men that received all radiation dose directed to the prostate, therefore men were excluded if the pelvis was included in the initial treatment volume. Men were excluded if protons or brachytherapy was used for radiation treatment. We then reviewed total radiation dose and excluded low doses that were clearly outliers from standard accepted doses during that time interval, range 35–50 Gy for SBRT and 72–86.4 Gy for IMRT. Patients with missing variables were then excluded, leaving 33,638 patients (Figure 1-CONSORT diagram).

Patients were then matched using propensity score matched (PSM) between treatment groups (IMRT or SBRT). The primary endpoint was overall survival (OS).

Demographic variables evaluable from the NCDB and matched by PSM include year of diagnosis, age, race, insurance status, residence location, median household income, patient comorbidity *via* the Charlson–Deyo comorbidity score, facility type, and treatment facility volume (divided into tertiles). Tumor and treatment specific factors evaluable from the NCDB include prostate-specific antigen (PSA), T stage, and Gleason score as well as use of androgen deprivation therapy. Radiation treatment dose was also stratified into low, medium, and high categories to be sure varying dose levels were evenly distributed between treatment groups. IMRT doses were defined as 7,200–7,559 cGy for low, 7,560–7,799 cGy for medium, and 7,800–8,640 cGy high. SBRT doses were defined as 3,500–3,624 cGy for low, 3,625–3,750 cGy for medium, and 3,751–5,000 cGy for high. All other doses below or above these defined categories were excluded.

Age was stratified into six groups (<55, 55–59, 60–64, 65–69, 70–74, 75–90 years). Race was characterized as either African–American, white, others, and unknown. Insurance status was outlined by the NCDB into six categories (Medicaid, Medicare, not insured, insurance status unknown, other governmental insurance, private insurance). The NCDB labeled residence location as metropolitan, rural, or urban using published files by the US Department of Agriculture Economic Research Service. Median household income was divided into quartiles <38K, 38–47,999, 48–62,999, +63, and unknown using average county level data from patient zip codes. Patient co-morbidities were coded as Charlson–Deyo comorbidity scores 0, 1, ≥2 (7). Type of cancer facility included academic/research programs, community cancer programs, comprehensive community cancer programs, integrated network cancer programs, and other. The NCDB used the American Joint Committee on Cancer Staging Atlas, sixth, and seventh edition for staging as appropriate for year of diagnosis.

Treatment groups were compared on demographic and clinical characteristics using χ^2^ test statistics. Propensity score 1–1 nearest neighbor matching without replacement was used to match treatment groups. Absolute standard mean differences (ASMDs) were used as a balance statistic for individual covariates, where an ASMD below 0.20 is desirable for all variables. Patients in the IMRT group were well matched with patients in the SBRT group on the following characteristics: age, race, residence, insurance status, median household income, Charlson–Deyo comorbidity scores, treatment facility type, year of diagnosis, tumor stage, PSA, and Gleason score. Scores calculated were blinded from researchers with respect to patient outcomes. OS was calculated from date of diagnosis to date of death or last follow-up. OS was estimated using Kaplan–Meier methodology, forming the basis of survival curves, and univariate comparisons were accomplished using log-rank test statistics. Propensity score matching was conducted using the MatchIt package in R version 3.30. All other statistical analyses were performed using SAS version 9.4 (SAS Inc., Cary, NC, USA).

This study was approved and carried out in accordance with the recommendations of the NCDB which provided a de-identified file for investigator use. The NCDB is not responsible for the analytical methodology or conclusions of the investigator.

## Results

### Patient Matching

**Figure 2:**
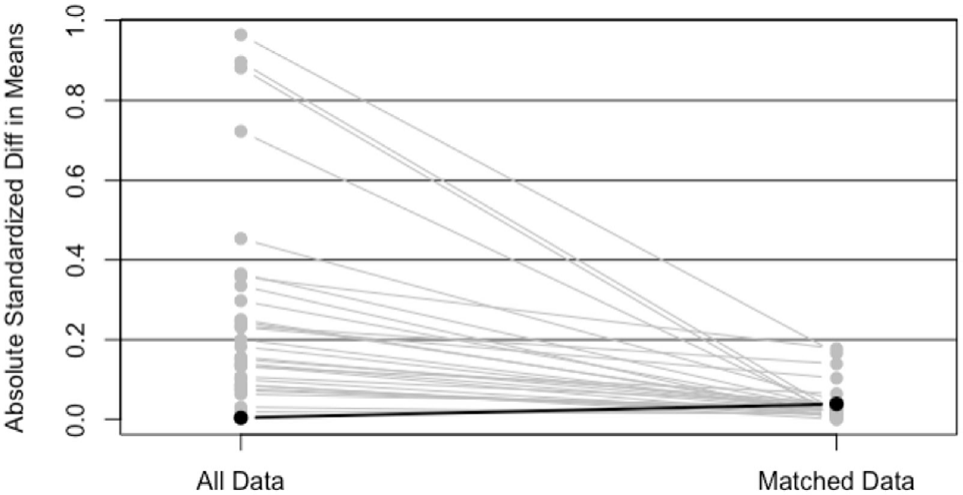
Plot of absolute standard mean differences (ASMDs) for original data and after 1–1 nearest neighbor PS matching.

Simple 1–1 nearest neighbor matching resulted in a matched sample of 5,430 with 2,715 in each group. Since these groups were well matched on the basis of ASMDs below 0.2, comparisons between treatment groups (IMRT, SBRT) could be made using a Kaplan–Meier curve, and a log-rank test statistic (Figure 2). The *p*-value corresponding to the log-rank test was above 0.05 (*p* = 0.6483), indicating that no significant differences between treatment groups were observed after matching.

### Patient Characteristics

A total of 5,430 men were included in the analysis after applying inclusion criteria, exclusion criteria, and performing PSM. There were 2,715 patients (50%) treated with SBRT and 2,715 patients (50%) treated with IMRT. Survival was evaluable through 8 years on the basis of an adequate number of patients at risk. Patient and treatment characteristics by treatment group are provided in Table 1.

**Table 1:** Population characteristics of matched sample by treatment group.

Characteristic	All patients *N* = 5,430 *N* (%)	IMRT *N* = 2,715 *N* (%)	SBRT *N* = 2,715 *N* (%)	*p*-Values
Age				0.4032
<55	275 (5.1%)	123 (4.5%)	152 (5.6%)	
55–59	518 (9.5%)	260 (9.6%)	258 (9.5%)	
60–64	1,411 (25.9%)	438 (16.1%)	454 (16.7%)	
65–69	1,276 (23.5%)	715 (26.3%)	696 (25.6%)	
70–74	1,058 (19.5%)	658 (24.2%)	618 (22.8%)	
75–90	1,058 (19.5%)	521 (19.2%)	537 (19.8%)	
Race				0.4803
Black	597 (11.0%)	281 (10.4%)	316 (11.6%)	
Other	79 (1.5%)	38 (1.4%)	41 (1.5%)	
Unknown	40 (0.7%)	20 (0.7%)	20 (0.7%)	
White	4,714 (86.8%)	2,376 (87.5%)	2,338 (86.1%)	
Insurance status				0.9208
Insurance status unknown	59 (1.1%)	31 (1.1%)	28 (1.0%)	
Medicaid	50 (0.9%)	24 (0.9%)	26 (1.0%)	
Medicare	3,289 (60.6%)	1,653 (60.9%)	1,636 (60.3%)	
Not insured	56 (1.0%)	30 (1.1%)	26 (1.0%)	
Other government	52 (1.0%)	23 (0.9%)	29 (1.1%)	
Private insurance	1,924 (35.4%)	954 (35.1%)	971 (35.7%)	
Patient residence				0.5233
Metropolitan	4,894 (90.1%)	2,442 (89.9%)	2,452 (90.3%)	
Rural	48 (0.9%)	21 (0.8%)	27 (1.0%)	
Urban	488 (9.0%)	252 (9.3%)	236 (8.7%)	
Median household income (US$)				0.0746
<38,000	451 (8.3%)	214 (7.9%)	237 (8.7%)	
38,000–47,999	773 (14.2%)	394 (14.5%)	379 (14.0%)	
48,000–62,999	1,223 (22.5%)	647 (23.8%)	576 (21.2%)	
63,000+	2,983 (54.9%)	1,460 (53.8%)	1,523 (56.1%)	
Charlson–Deyo comorbidity score				0.0155
0	4,783 (88.1%)	2,417 (89.0%)	2,366 (87.2%)	
1	555 (10.2%)	247 (9.1%)	308 (11.3%)	
2	92 (1.7%)	51 (1.9%)	41 (1.5%)	
Facility type				0.0026
Academic/research program	2,660 (49.0%)	1,269 (46.7%)	1,391 (51.2%)	
Community cancer program	55 (1.0%)	27 (1.0%)	28 (1.0%)	
Comprehensive community program	2,291 (42.2%)	1,214 (44.7%)	1,077 (39.7%)	
Integrated network cancer program	424 (7.8%)	205 (7.6%)	219 (8.1%)	
Year of diagnosis				0.5820
2004–2009	2,266 (41.7%)	1,123 (41.4%)	1,143 (42.1%)	
2010–2013	3,164 (58.3%)	1,592 (58.6%)	1,572 (57.9%)	
Tumor clinical stage				0.8034
Other	52 (1.0%)	24 (0.9%)	28 (1.0%)	
T1	4,333 (79.8%)	2,180 (80.3%)	2,153 (79.3%)	
T2	1,027 (18.9%)	502 (18.5%)	525 (19.3%)	
T3	18 (0.3%)	9 (0.3%)	9 (0.3%)	
Prostate-specific antigen				0.2692
<10	4,455 (82.0%)	268 (9.9%)	289 (10.6%)	
10–20	557 (10.3%)	2,250 (82.9%)	2,205 (81.2%)	
>20	418 (7.7%)	197 (7.3%)	221 (8.1%)	
Gleason score				0.0182
5	32 (0.6%)	13 (0.5%)	19 (0.7%)	
6	3,020 (55.6%)	1,558 (57.4%)	1,462 (53.9%)	
7	2,087 (38.4%)	990 (36.5%)	1,097 (40.4%)	
8	214 (3.9%)	107 (3.9%)	107 (3.9%)	
9	73 (1.3%)	45 (1.7%)	28 (1.0%)	
10	4 (0.1%)	2 (0.1%)	2 (0.1%)	
Hormone therapy				0.4834
No	4,925 (90.7%)	2,455 (90.4%)	2,470 (91.0%)	
Yes	505 (9.3%)	260 (9.6%)	245 (9.0%)	
Dose level				<0.0001
Low	1,076 (19.8%)	436 (16.1%)	640 (23.6%)	
Intermediate	3,903 (71.9%)	2,039 (75.1%)	1,864 (68.7%)	
High	451 (8.3%)	240 (8.8%)	211 (7.8%)	

No significant differences were observed in the distributions for age, race, insurance status, patient residence, median household income, year of diagnosis, clinical tumor stage, PSA, or hormone therapy between the matched treatment groups on the basis of *p* < 0.05. Significant differences in matched groups existed on the following variables: Charlson–Deyo score (*p* = 0.0155), facility type (*p* = 0.0026), Gleason score (*p* = 0.0182), and dose category (*p* < 0.0001).

### OS for Matched Patients

**Figure 3:**
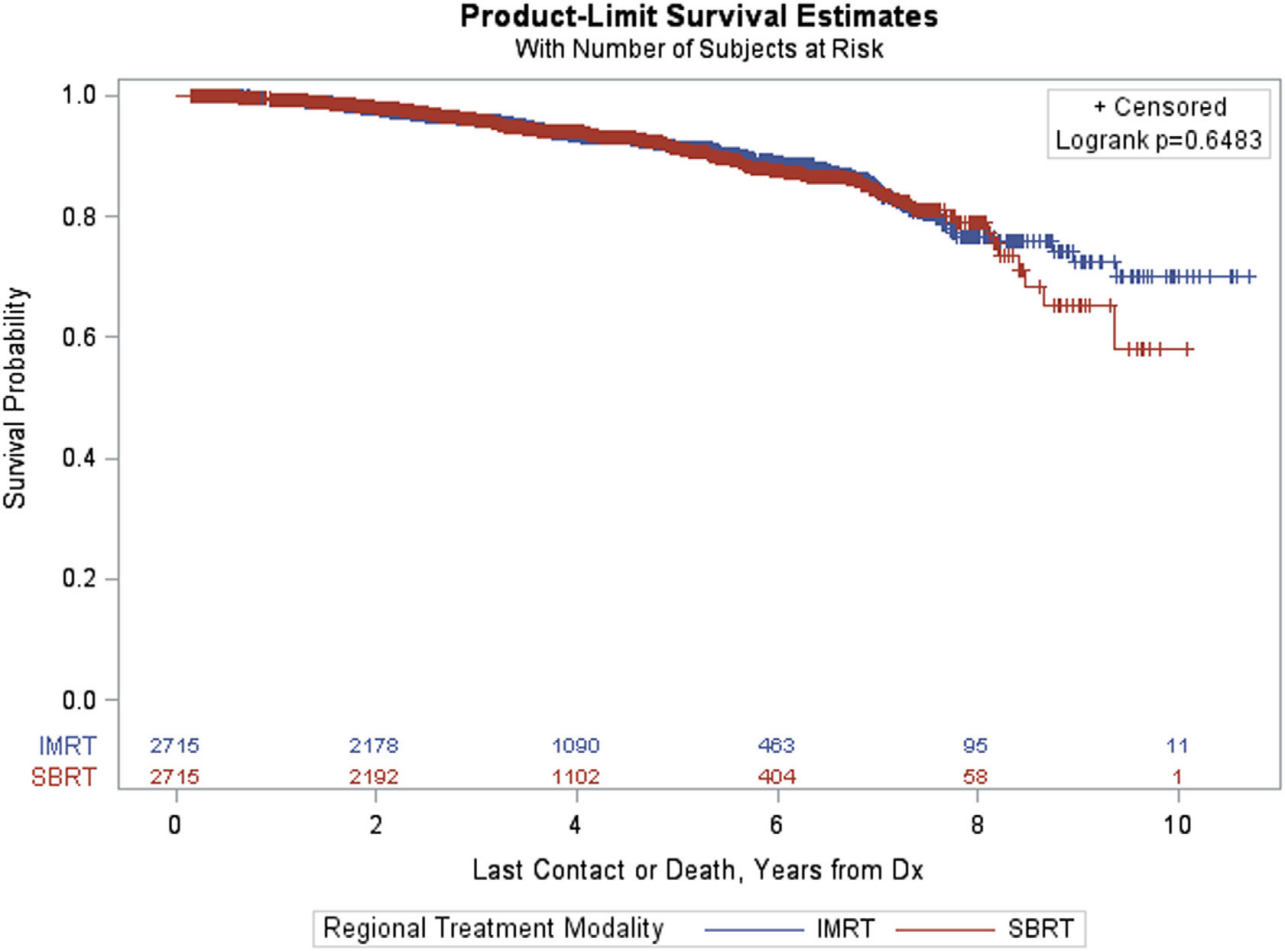
Kaplan–Meier estimates by treatment group in propensity score matched sample.

**Figure 4:**
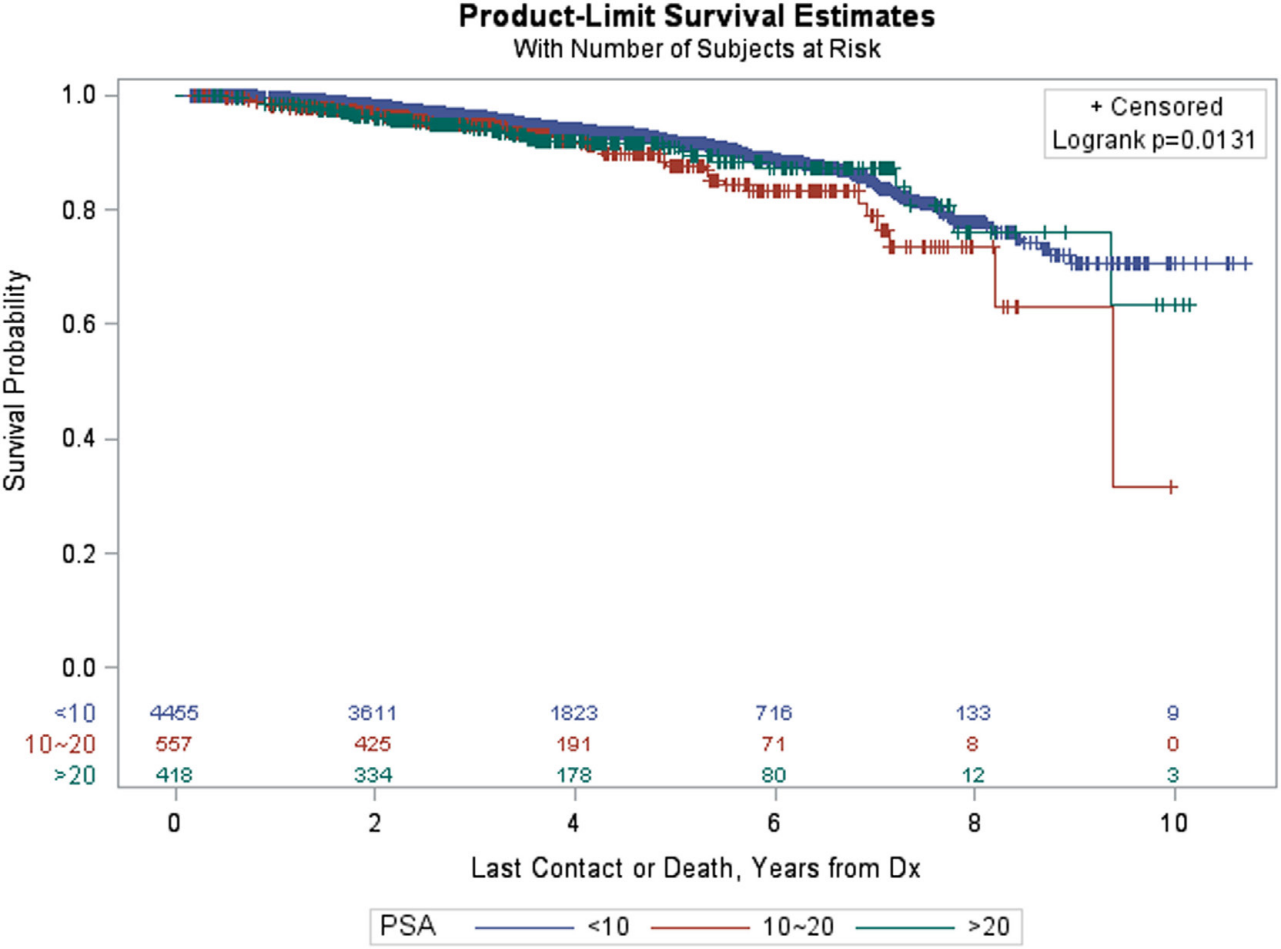
Kaplan–Meier estimates by prostate-specific antigen in propensity score matched sample.

**Figure 5:**
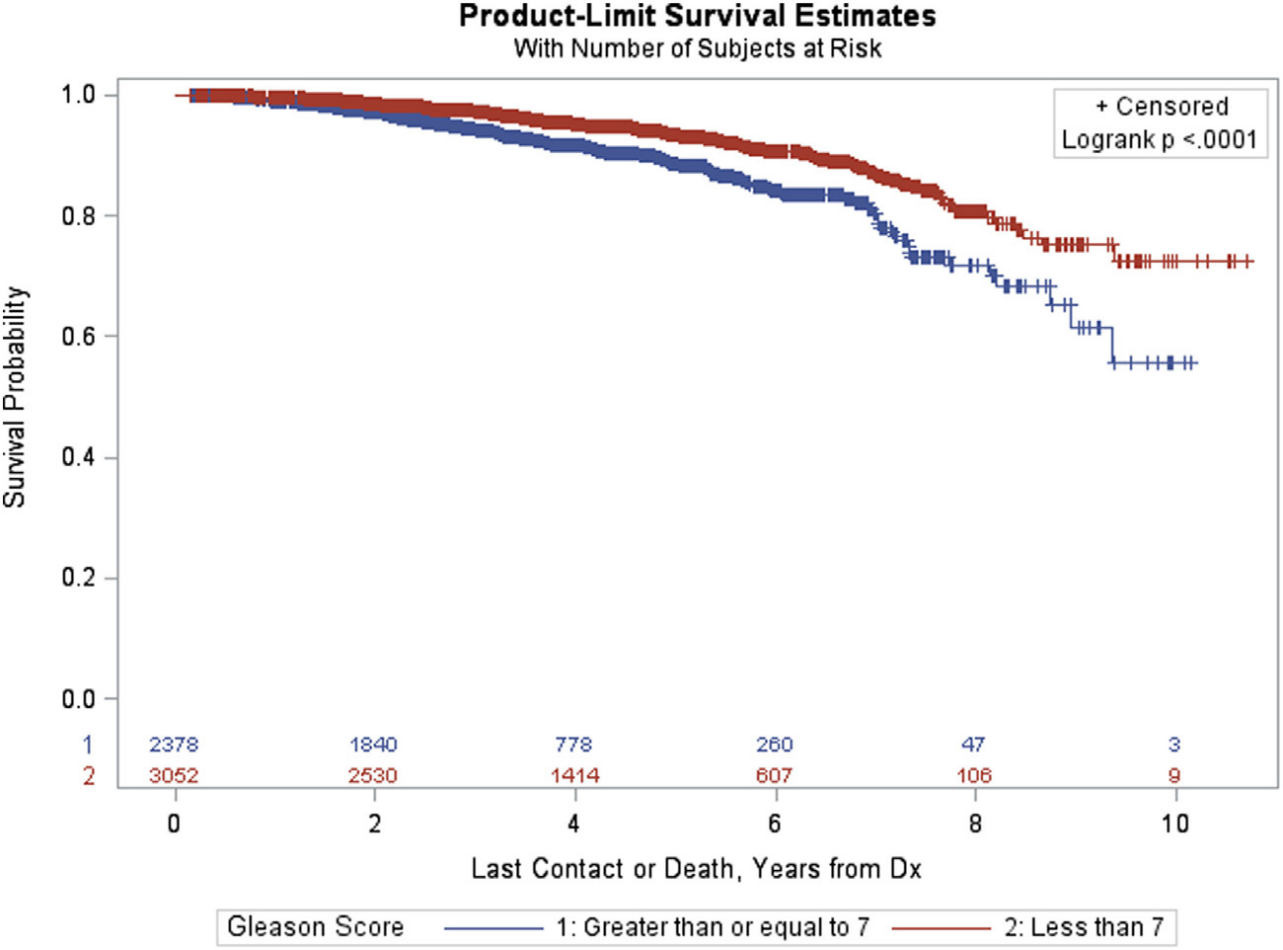
Kaplan–Meier estimates by Gleason score in propensity score matched sample.

Figure 3 provides Kaplan–Meier OS estimates by treatment group. No statistically significant overall differences are observed (*p* = 0.6483), and the estimated OS at 8 years for IMRT and SBRT patients was 77.23 and 79.38%, respectively. The estimated OS at 8 years for other patient variables, both within the matched and pre-matched groups, are provided in Table 2. Age (*p* < 0.0001), insurance status (*p* = 0.0002), patient residence (*p* = 0.0145), median household income (*p* = 0.0059), Charlson–Deyo comorbidity score (*p* < 0.0001), PSA (*p* = 0.0131, Figure 4), and GS (*p* < 0.0001, Figure 5) demonstrated statistically significant differences in OS on univariate analysis (UVA) for the matched group. All other factors on UVA did not demonstrate statistically significant differences.

**Table 2:** Estimated KM overall survival at 8 years for all variables.

Variable	Matched sample (*N* = 5,430)		Whole sample (*N* = 33,638)	
	% survived at 8 years	Log-rank *p*-value	% survived at 8 years	Log-rank *p*-value
Treatment		0.6483		0.0056
IMRT	77.23		75.50	
SBRT	79.38		79.38	
Age		<0.0001		<0.0001
<55	98.60		87.42	
55–59	79.63		85.85	
60–64	93.47		84.44	
65–69	81.85		80.26	
70–74	78.35		76.24	
75–90	59.77		63.73	
Race		0.2813		0.0003
Black	83.99		75.96	
Other	71.87		79.84	
Unknown	100.00		84.05	
White	77.60		75.38	
Insurance status		0.0002		<0.0001
Insurance status unknown	78.28		77.01	
Medicaid			69.54	
Medicare	74.41		72.21	
Not insured	86.60		76.88	
Other government	89.84		77.71	
Private insurance	84.80		83.35	
Patient residence		0.0145		0.0599
Metropolitan	79.45		76.14	
Rural	61.00		69.61	
Urban	63.89		73.41	
Median household income (US$)		0.0059		<0.0001
<38,000	78.13		72.58	
38,000–47,999	63.15		73.25	
48,000–62,999	81.35		75.64	
63,000+	80.88		78.48	
Charlson–Deyo comorbidity score		<0.0001		<0.0001
0	79.32		77.22	
1	73.73		64.39	
2	33.20		53.74	
Facility type		0.9955		<0.0001
Academic/research program	75.94		77.74	
Community cancer program	67.70		72.41	
Comprehensive community program	80.36		74.75	
Integrated network cancer program	75.57		77.35	
Year of diagnosis		0.0210		0.0118
2004–2009	78.69		75.86	
2010–2013				
Tumor clinical stage		0.0826		<0.0001
Other	90.44		76.45	
T1	78.69		77.15	
T2	74.57		73.28	
T3	80.82		65.30	
Prostate-specific antigen		0.0131		<0.0001
<10	78.65		77.69	
10–20	72.72		69.27	
>20	77.61		70.62	
Gleason score		<0.0001		<0.0001
<7	81.35		80.65	
≥7	71.96		70.85	
Hormone therapy		0.0020		<0.0001
No	78.49		78.19	
Yes	73.05		70.97	
Dose level		0.5403		0.1488
Low	76.93		75.77	
Intermediate	77.86		75.15	
High	86.18		77.22	

### OS by Subpopulation

In order to further characterize the effect of treatment modality among higher risk prostate cancer patients, three additional PSM analyses (1–1 nearest neighbor matching without replacement) were carried out within patients having a pretreatment PSA > 10 or GS > 7 or GS = 7.

**Figure 6:**
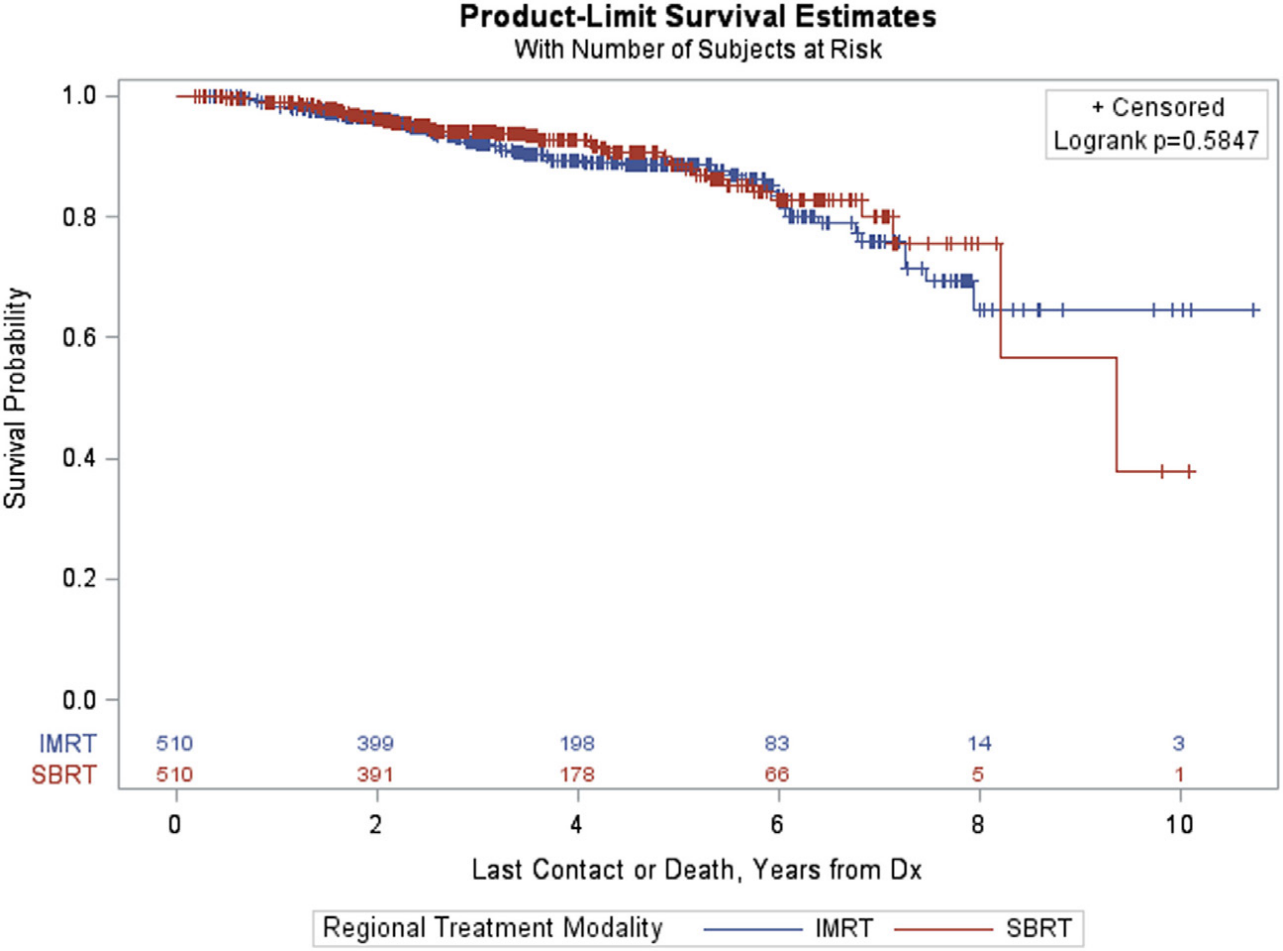
Kaplan–Meier estimates by treatment group in propensity score matched sample of prostate-specific antigen > 10 patients.

The PSM analysis of patients with PSA > 10 resulted in a matched sample of 1,020 (510 in each group). The groups were well matched on the basis of ASMD values below 0.20, and thus differences between treatment groups (IMRT vs SBRT) could be carried out using a Kaplan–Meier curve and a log-rank test (Figure 6). The *p*-value corresponding to the log-rank test was again above 0.05 (*p* = 0.5847), indicating that there were no statistically significant differences between treatment groups after matching.

**Figure 7:**
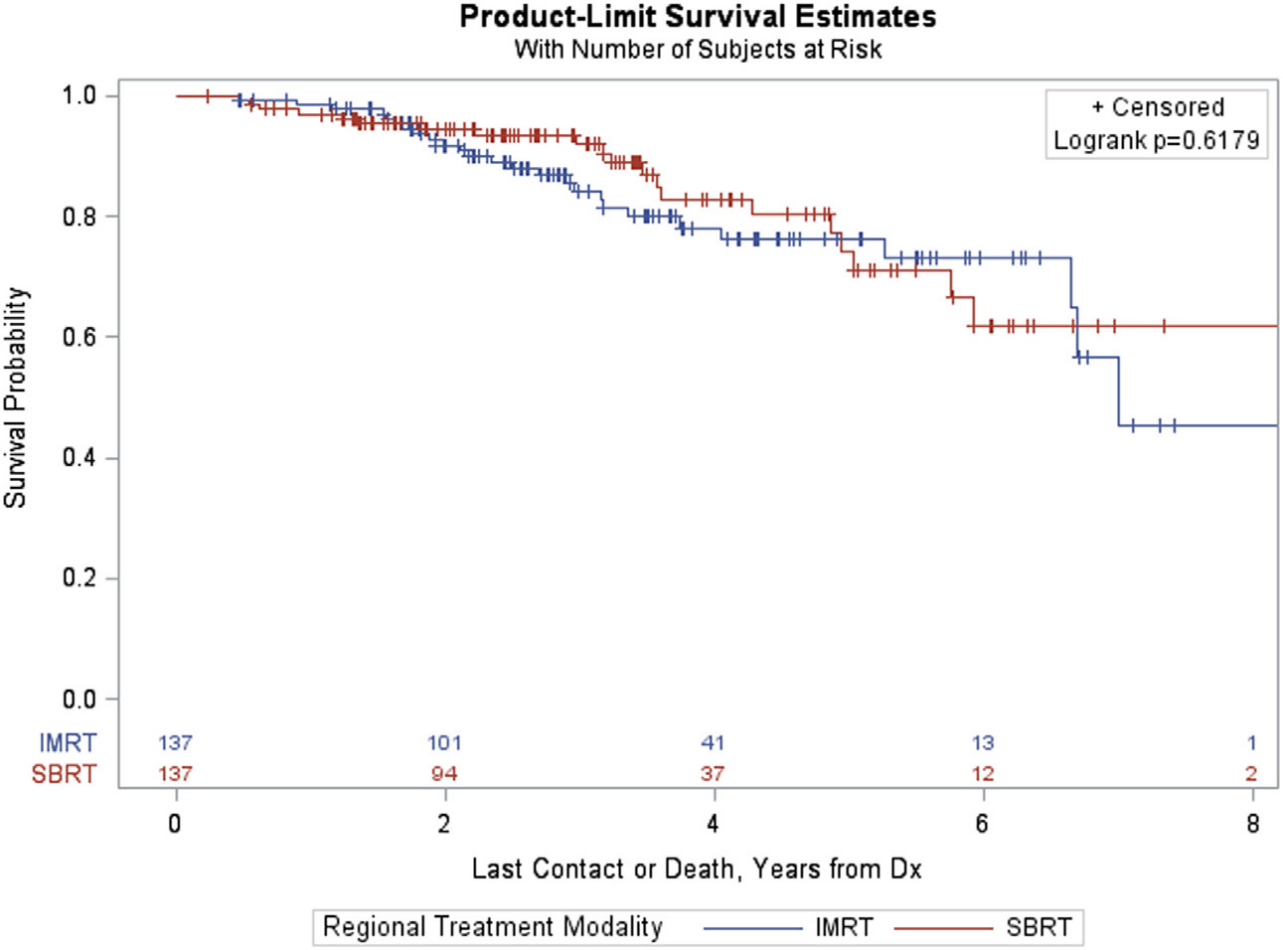
Kaplan–Meier estimates by treatment group in propensity score matched sample of GS > 7 patients.

Among patients with GS > 7, PSM resulted in a matched sample of 274 (137 in each group). Again, the groups were well matched and differences between treatment groups (IMRT vs SBRT) resulted in a non-significant log-rank *p*-value (*p* = 0.6179, Figure 7).

**Figure 8:**
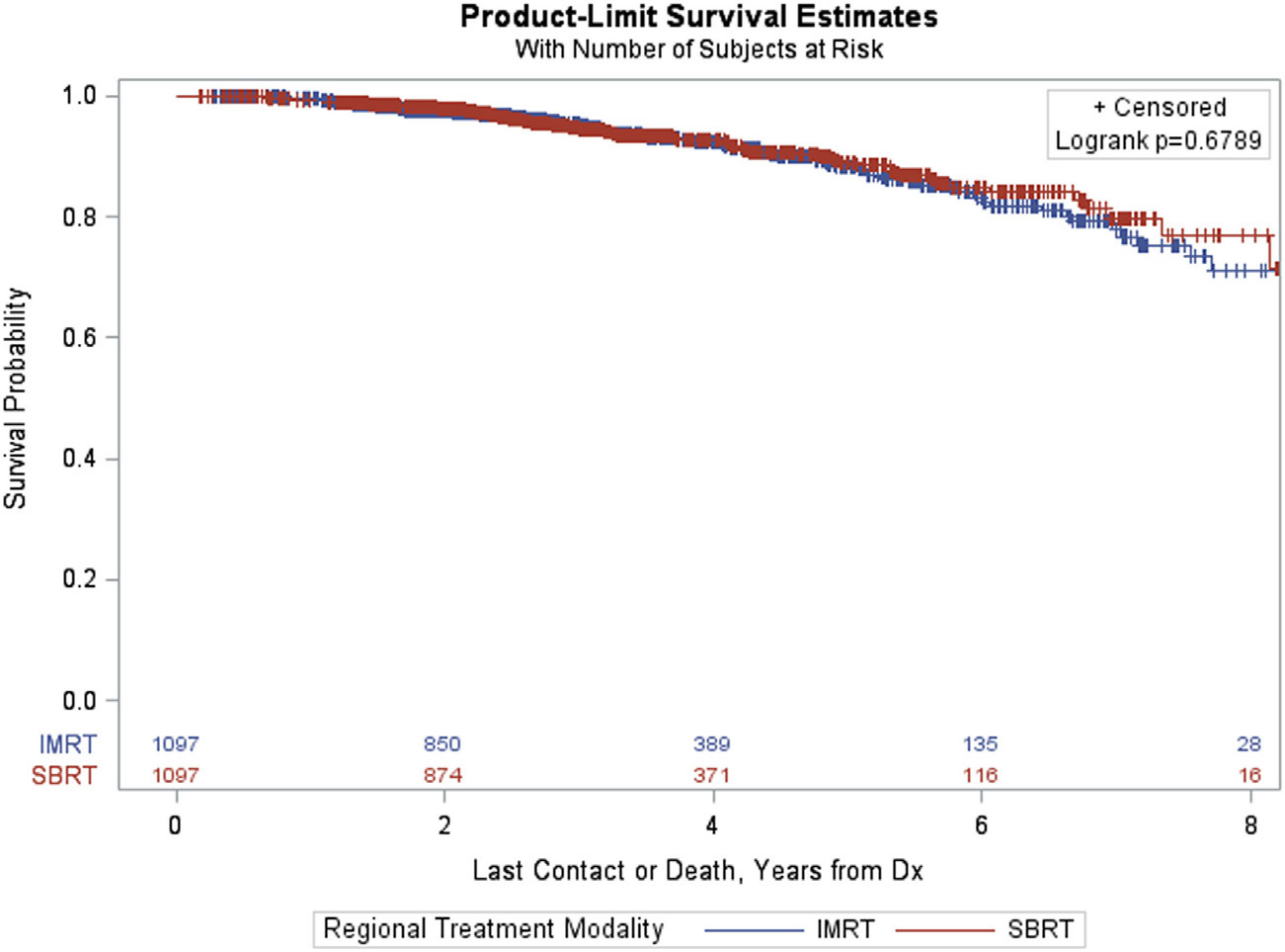
Kaplan–Meier estimates by treatment group in propensity score matched sample of GS = 7 patients.

Finally, the PSM analysis of patients with GS = 7 resulted in a matched sample of 2,194 (1,097 in each group). The groups were well matched and differences between treatment groups (IMRT vs SBRT) resulted in a non-significant log-rank *p*-value (*p* = 0.6789, Figure 8).

## Discussion

No significant difference in survival between SBRT and IMRT for localized prostate cancer was found utilizing the NCDB with PSM matching at 8 years. In addition, we found no significant difference in OS between the two treatment modalities in matching high-risk subpopulations of GS = 7 or GS > 7 or PSA > 10. As expected, differences in OS by patient and clinical characteristics were observed among men with older age, higher comorbidity score, higher GS, and higher PSA.

Patient demographics and treatment characteristics in both treatment groups showed some statistically significant differences that were not controlled by PSM. These differences include two variables that significantly impacted survival in this study: Charlson–Deyo comorbidity score and GS. When comparing comorbidity scores, although not necessarily clinically significant, there are an increased proportion of “healthy” patients (comorbidity score = 0) in the IMRT group vs the SBRT group (89.0 vs 87.2%, respectively). In addition, the SBRT group has a higher proportion of patients with comorbidity score = 1 (11.3 vs 9.1%, respectively). These results could potentially add bias against the SBRT treatment group, which appears to have worse comorbidity scores. The differences seen in GS distribution could also potentially bias against SBRT, with a greater proportion of GS = 7 (40.4 vs 36.5%) and a lower proportion of GS = 6 (53.9% vs 57.4%).

The strength of the current study is the large number of patients allowing for 8-year survival estimates by known risk factors for prostate cancer as well as other demographic and treatment factors not normally evaluated in single institutional or randomized trials. This database is homogeneous with regard to treatment technique with only men treated to the prostate with IMRT or SBRT analyzed. The database is homogeneous with regard to dose with stratification by low-, intermediate-, and high- dose groups for matching. Patients with no follow- up or outliers with regard to dose were excluded.

A weakness of this study is that survival is the only outcome available—specifically, there is no biochemical or toxicity information. With the 2017 NCCN risk stratification, however, survival is the most important outcome parameter with treatment recommended only if at least 10-year survival is expected based on medical status. The NCDB potentially includes selection bias since only accredited hospitals input data which could represent patients receiving higher quality care. As such this analysis may not be a true population-based study which limit’s the study generalizability (8, 9).

Intensity modulated radiation therapy continues to be a standard radiotherapeutic technique for the definitive treatment of localized prostate cancer using a conventionally fractionated approach with 1.8–2.0 Gy fractions 5 days per week over 8–9 weeks. A decade of data supports this fractionation scheme for localized prostate cancer, with excellent biochemical control, OS, and acceptable toxicity. Dose-escalation trials revealed improved biochemical freedom from relapse (BFFR) in certain trials but no difference in survival as updated in a 2015 systematic review (1, 10–14).

Moderate hypofractionated radiotherapy for prostate cancer has been studied in six phase III randomized trials with varied number of fractions (19–30) and doses (52.5–72 Gy). Two of the trials were carried out before dose-escalation was known and therefore delivered insufficient dose (15, 16). Three of the trials showed no significant difference reported in BFFR, OS, or toxicity compared with conventional fractionation for prostate cancer with median follow-up from 51 to 90 months (17–19). The recent 5-year data published from the non-inferiority HYPRO trial of intermediate- and high-risk patients concluded that dose-escalated moderate hypofractionation was not superior with comparable relapse-free survival but higher toxicity profiles compared with standard fractionation (20, 21).

Single institutions, pooled institutions, and registries have shown similar efficacy and toxicity of SBRT compared with IMRT in low and select intermediate-risk prostate cancer (22–25). The study with longest follow-up was published in abstract form for mostly low- and intermediate-risk prostate cancer patients with 9-year FFBF of 95% low-risk, 89% intermediate-risk, and 66% high-risk groups. Extreme hypofractionation was tolerated well with 1.9% RTOG grade 3 urinary toxicity but no grade 3 GI toxicity (4).

Several trials will address the remaining questions regarding biochemical, toxicity, and survival outcomes for extreme hypofractionation. RTOG 0938, an equivalency study of low-risk prostate cancer, randomized extreme hypofractionation 36.25 Gy in 5 fractions to moderate hypofractionation of 51.6 Gy in 12 fractions. The study was closed February 2014 with 255 patients accrued with quality of life at 1 year the primary outcome. It was recently published that both the 5 fraction and 12 fraction regimens were well tolerated (26). A recent dose-escalation trial for prostate cancer treated with SBRT has also shown acceptable toxicities up to 47.5 Gy over 2.5 weeks (27). Three randomized trials await completion comparing conventional fractionation or moderate hypofractionation to extreme hypofractionation (28–30).

The Technology Assessment produced by the Agency for Healthcare Research and Quality, “Comparative Evaluation of radiation treatments for clinically localized prostate cancer: an update,” analyzed 60 high-quality studies including 9 RCTs, and determined that there is insufficient evidence to support either SBRT vs IMRT, noting that there was no high-quality study comparing SBRT to any other radiation modality. The Institute of Medicine has also included prostate cancer comparative effectiveness research in the “top quartile” group for priority (31). This NCDB PSM analysis for clinically localized prostate cancer compares these two radiation treatment modalities with a large sample size and provides evidence to suggest no difference in OS through 8 years.

## Conclusion

In a PSM analysis of the NCDB, no difference in OS was observed when comparing IMRT to SBRT in the treatment of localized prostate cancer. Subset analyses of intermediate- and high-risk patients (Gleason score = 7 or >7 or PSA > 10) confirmed no observed difference in OS by treatment within these populations. We await randomized data to confirm these survival findings.

## Ethics Statement

Ethics statements were placed in the body of Section “Materials and Methods” in the manuscript.

## Author Contributions

All authors contributed to the conception and design, analysis, interpretation of data, drafting of the abstract, and its revision for important intellectual comment.

## Conflict of Interest Statement

RL is a partial stockholder of Philadelphia Cyberknife. All authors have read and approved the manuscript. We have no financial disclosures. We are not using any copyrighted information, patient photographs, identifiers, or other protected health information in this paper. No text, text boxes, figures, or tables in this article have been previously published or owned by another party.
